# The DOCK Protein Sponge Binds to ELMO and Functions in *Drosophila* Embryonic CNS Development

**DOI:** 10.1371/journal.pone.0016120

**Published:** 2011-01-25

**Authors:** Bridget Biersmith, Ze Liu, Kenneth Bauman, Erika R. Geisbrecht

**Affiliations:** 1 Division of Cell Biology and Biophysics, School of Biological Sciences, University of Missouri, Kansas City, Missouri, United States of America; 2 Ph.D. Program, School of Biological Sciences, University of Missouri, Kansas City, Missouri, United States of America; Skirball Institute of Biomolecular Medicine - New York University Medical Center, United States of America

## Abstract

Cell morphogenesis, which requires rearrangement of the actin cytoskeleton, is essential to coordinate the development of tissues such as the musculature and nervous system during normal embryonic development. One class of signaling proteins that regulate actin cytoskeletal rearrangement is the evolutionarily conserved CDM (*C. elegans* Ced-5, human DOCK180, *Drosophila* Myoblast city, or Mbc) family of proteins, which function as unconventional guanine nucleotide exchange factors for the small GTPase Rac. This CDM-Rac protein complex is sufficient for Rac activation, but is enhanced upon the association of CDM proteins with the ELMO/Ced-12 family of proteins. We identified and characterized the role of *Drosophila* Sponge (Spg), the vertebrate DOCK3/DOCK4 counterpart as an ELMO-interacting protein. Our analysis shows Spg mRNA and protein is expressed in the visceral musculature and developing nervous system, suggesting a role for Spg in later embryogenesis. As maternal null mutants of *spg* die early in development, we utilized genetic interaction analysis to uncover the role of Spg in central nervous system (CNS) development. Consistent with its role in ELMO-dependent pathways, we found genetic interactions with *spg* and *elmo* mutants exhibited aberrant axonal defects. In addition, our data suggests Ncad may be responsible for recruiting Spg to the membrane, possibly in CNS development. Our findings not only characterize the role of a new DOCK family member, but help to further understand the role of signaling downstream of N-cadherin in neuronal development.

## Introduction

The formation of embryonic tissues is a key feature in generating diversity in animal development. After cell fate is established, cell-cell signaling and intracellular signal transduction pathways instruct cells to undergo cell shape changes. These cell shape changes are necessary for cell movement, a basic process that underlies embryonic development and is largely accomplished by regulation of the actin cytoskeleton. Actin dynamics is required for the migration of individual groups of cells, as in border cell migration in the *Drosophila* ovary, or large groups of cells, such as those involved in gastrulation in the developing fly embryo [1], [2]. One common feature of cell rearrangements via the actin cytoskeleton is the involvement of the Rho family of GTPases [3], [4].

Widely conserved across species and involved in seemingly diverse developmental processes including cell migration, phagocytosis, and myoblast fusion, the Rho GTPases are key signaling molecules that impinge upon actin cytoskeletal reorganization [5]. Several classes of GTPase regulatory proteins have been identified, including the GTPase-activating proteins (GAPs), guanine nucleotide exchange factors (GEFs), and guanine nucleotide dissociation inhibitors (GDIs) [6], [7]. In particular, the GEFs regulate GTPase activity by exchanging the inactive, GDP-bound Rac to the active, GTP-bound state. It is thought that GEFs are a crucial intermediate that signal from upstream cell surface receptors to mediate GTPase activation. Some GEFs directly associate with membrane receptors, while others are associated via an intermediate complex. In flies, two neuronally expressed Rac GEFs have been identified that exemplify this in development of the central nervous system. Trio physically interacts with the Netrin receptor Frazzled to regulate chemoattraction [8], [9], while Son of sevenless (Sos) associates with the Roundabout (Robo) receptor through the SH2-SH3 adaptor protein Dreadlocks (DOCK) to control axon repulsion [9].

Recent studies have identified a class of non-canonical GEFS that are members of the CDM (*C. elegans* Ced-5, human DOCK180, *Drosophila* Myoblast city) family of proteins [5], [10]. Evolutionarily conserved, Mbc/DOCK180/Ced-5 proteins contain an N-terminal Src-homology-3 domain (SH3), two internal DOCK-homology regions (DHR-1 and DHR-2), and a C-terminal proline–rich region. The DHR1 regions of both DOCK180 and Mbc bind to phosphatidylinositol 3,4,5-triphosphate [PtdIns(3,4,5)P_3_] [11], [12]. Vertebrate cell culture studies show this region is required for membrane localization [12]. In flies, the DHR1 domain is not essential for recruitment to the membrane, but is essential for myoblast fusion as deletion of the DHR1 domain fails to rescue *mbc* mutant embryos in functional rescue assays [11]. Although the SH3-domain containing protein Crk is capable of binding the C-terminal proline-rich region of both DOCK180 and Mbc, it is not always essential *in vivo*. A direct interaction between vertebrate DOCK180 and CrkII is not required for apoptotic cell removal [13]. Furthermore, deletion of the Ced-2/Crk binding sites in *C. elegans* Ced-5/DOCK180 does not affect cell engulfment or migration [13]. Consistent with this, while *Drosophila* Crk binds Mbc, it is dispensable for myoblast fusion [11]. Whereas canonical GEFs contain both typical Dbl-homology domain (DH) and Pleckstrin-homology domains (PH) that are involved in activation of the Rho GTPases, these domains are absent in CDM family members [10], [12]. Conventional GEFs bind nucleotide-free Rac via their DH domain, while the CDM proteins use the DHR2 region. Deletion or mutation of this domain results in a loss of Rac binding and activation [14], [15]. A DOCK-Rac protein complex is sufficient for Rac activation [12], [16], but may be enhanced by DOCK180 bound to ELMO [14], [17], [18].

ELMO/Ced-12 (hereafter referred to as ELMO) was originally identified in *C. elegans* as an upstream regulator of Rac in apoptotic cell engulfment and cell migration [19], [20], [21]. Studies using mammalian ELMO1 subsequently showed that the DOCK180-ELMO complex is required for Rac-mediated cell migration and phagocytosis [14], [17], [18], [22], [23]. The PH domain, which in conventional GEFs targets protein to the membrane through its interactions with phosphatidylinositol lipids or other protein-protein interactions, is provided by the ELMO protein in the DOCK-ELMO complex [14], [16]. The N-terminal SH3 domain of CDM family members associates with the C-terminal region of the ELMO family of proteins [24]. While the molecular function of ELMO in the DOCK→Rac signaling pathway still needs to be clarified, it is worth noting that ELMO has functions independent of the DOCK proteins.

Importantly, studies in *Drosophila* have provided additional insight into role of the Mbc-ELMO→Rac signaling pathway in multiple tissues. Mutations in *mbc* and *elmo* result in border cell migration defects in the ovary and myoblast fusion defects in the embryo [25], [26], [27]. Decreased Mbc and ELMO function exhibit abnormal ommatididal organization in the eye and thorax closure defects in the adult [27], [28]. In addition, loss-of-function studies have demonstrated that the *Rac* genes are required redundantly in a variety of developmental processes, including border cell migration, myoblast fusion, and axon guidance in the developing nervous system [27], [29], [30], [31]. Last, genetic interactions exist between the atypical GEF Mbc-ELMO complex and their target GTPase Rac. A genetic screen in the eye uncovered an allele of *mbc* that suppresses the Rac1 overexpression phenotype [32]. In support of this, removal of one copy of both *Rac1* and *Rac2* are capable of ameliorating the “activated-Rac” phenotype exhibited by co-expression of both Mbc and ELMO in the eye [27].

Although the work cited above provides convincing evidence that the DOCK180/Mbc-ELMO complex is essential in development, the mechanism by which at least five Rac-specific DOCK proteins bind to one or more ELMO proteins in vertebrates to modulate actin regulation in a tissue-specific manner is not clear. DOCK180, DOCK4, and DOCK5 are broadly expressed in many tissues, including the brain and nervous system [33]. In contrast, DOCK2 is expressed specifically in hematopoietic cells, while DOCK3 expression is primarily restricted to the brain and spinal cord [34], [35], [36]. In addition to their complex expression patterns, DOCK family members exhibit pleiotropic functions in development. DOCK180 has recently been shown to be required for Rac-mediated axon outgrowth in cortical neurons in response to netrin-1, neurite outgrowth as mediated by nerve growth factor, and axon pruning via ephrin-B3 [17], [37], [38]. Mouse knock-outs show DOCK180 is required in concert with DOCK5 in muscle fusion [39]. DOCK3 (or modifier of cell adhesion, MOCA) colocalizes with N-cadherin and actin in neuronal differentiation [36], [40]. MOCA is also linked to Alzheimer's disease (AD), where it accumulates in neurofibrillary tangles and modulates beta-amyloid (APP) precursor processing [41], [42], [43]. Consistent with this, mice lacking DOCK3 exhibit axonal degeneration [44]. Finally, knockdown of DOCK4 results in reduced dendritic growth and branching in hippocampal neurons [45]. *Drosophila* provides an excellent system to characterize this conserved pathway with a single ELMO ortholog. Using proteomics approaches for identifying new players in the ELMO-mediated pathway in the developing embryo, we have uncovered Spg, the *Drosophila* ortholog of human DOCK3/4, as an ELMO-interacting protein. In contrast to the well-established role of Mbc in myoblast fusion, Spg is not required with ELMO in somatic muscle development. However, the two *Drosophila* DOCK family members Mbc and Spg are required in the developing nerve cord. Moreover, Spg can be recruited to the membrane by N-cadherin in S2 cells, providing a mechanism for Spg localization that may function to mediate the development of axonal pathways.

## Results

### Identification of the DOCK3 and DOCK4 ortholog CG31048/Sponge as an ELMO-interacting protein

**Figure 1:**
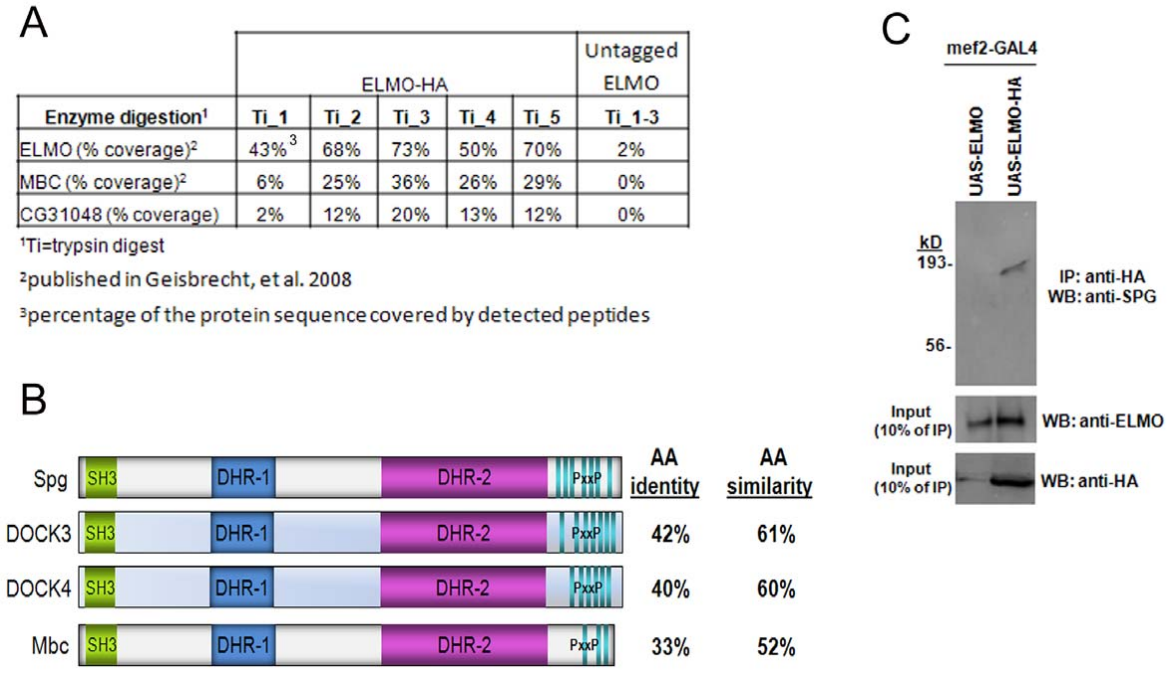
Identification of CG31048/Spg as an ELMO-binding protein. (A) Table showing peptide coverage of HA-tagged ELMO in 5 independent mass spectrometry experiments compared to 3 untagged ELMO control experiments. Aside from ELMO itself, the most abundant associated protein detected was Mbc, followed by CG31048. (B) Protein schematic of Spg and related proteins. Spg is the most similar to vertebrate DOCK3 and DOCK4. The most closely related fly protein is Mbc. SH3 (Src-homology domain-3); DHR-1 (DOCK Homology Region-1); DHR-2 (DOCK Homology Region-2); PxxP (Proline-rich region). (C) Both tagged and untagged ELMO are expressed under control of the muscle-specific *mef2-GAL4* driver. Embryonic lysates are immunoprecipitated with anti-HA and immunoblotted with antisera against Spg (top panel). Inputs show loading of total ELMO protein (middle panel) and HA-tagged protein (bottom panel).

To identify proteins that may interact with ELMO in the developing embryonic musculature, tissue-specific immunoprecipitations (IPs) were carried out as described in Geisbrecht, et al [27]. In brief, either HA-tagged or untagged ELMO, both of which rescue *elmo* mutants, were expressed using the muscle-specific *mef2*-*GAL4* driver. ELMO-specific complexes were isolated from embryonic lysates with anti-HA resin, digested with trypsin, and analyzed by Multidimensional Protein Identification Technology (MudPIT) mass spectrometry [46]. In an average of 5 independent experiments, the percent peptide coverage of ELMO ranged from 43–73% (Figure 1A), while the most abundant associated protein was Mbc [27]. Peptides corresponding to the protein CG31048 were detected in lysates immunoprecipitated with tagged ELMO, but not untagged ELMO. After Mbc, CG31048 was the second most abundant protein detected, where the percentage of peptide coverage that corresponded to CG30148 ranged from 2–30%. While the *CG31048* cDNA had not yet been cloned, an abstract from the 2005 fly meeting by Eyal Schejter, et al., linked this locus to a maternal effect mutant called *sponge* (*spg*), whose name we will use hereafter. An allele of *spg* was originally identified by Rice and Garen [47], while more alleles emerged from screens in the laboratory of C. Nusslein-Volhard. Postner, et al., examined the role of Spg in early actin cap and metaphase furrow formation in early embryonic development [48]. In addition, the Rorth lab determined that both Mbc and Spg function redundantly in border cell migration downstream of the receptor PVR [49]. However, the role of Spg in later embryonic processes has not been examined.

Spg is most closely related to both mammalian DOCK3/MOCA and DOCK4 and is a CDM family member whose domain structure is highly similar to Mbc (Figure 1B). All of these related proteins contain an N-terminal Src-homology 3 domain (SH3), and internal DOCK homology region-1 (DHR-1) and DOCK homology region-2 (DHR-2) domains. Spg shares greater amino acid sequence identity to vertebrate DOCK3 and DOCK 4 (42% and 40%, respectively) than Mbc (33%). This primary amino acid identity/similarity (33%/52%) between Spg and Mbc decreases to 16% amino acid identity and 21% amino acid in the C-terminal proline-rich region. Notably, the C-terminal region of Spg contains 7 predicted proline rich sites not present in Mbc. This is similar to vertebrate analyses of DOCK family members, where the number of proline-rich sites in the C-terminal region of DOCK3 and DOCK4 is greater than that found in DOCK180 alone [50]. It is hypothesized that this region may confer differential properties of DOCK family function.

To confirm a potential physical interaction between ELMO and CG31048, we generated antisera to the C-terminal region of Spg that is the most divergent from Mbc. Similar to the MS experiments in which Spg was identified, both HA-tagged ELMO and untagged ELMO were expressed in the developing musculature with *mef2-GAL4*. After preparing embryonic lysates, anti-HA beads were used to immunoprecipitate HA-tagged and untagged ELMO. Consistent with results that show both vertebrate DOCK3 and DOCK4 are associated with ELMO [23], [51], Spg could be visualized in an ELMO-associated complex by immunoblotting with anti-Spg (Figure 1C).

### Spg mRNA and protein is strongly expressed in the developing nervous system

**Figure 2:**
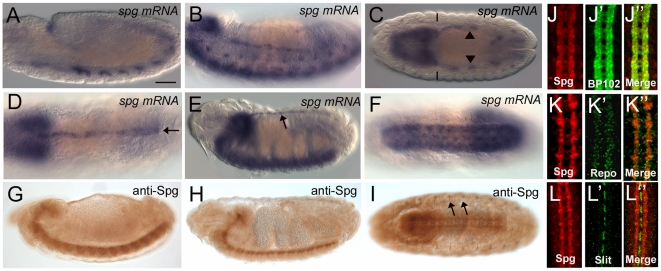
Spatial expression of Spg in the developing embryo. (A–F) *In situ* hybridizations of wild-type embryos showing *spg mRNA* expression. (A) Stage 11 embryo shows expression in the nervous system primordia. (B) Expression in the ventral nerve cord and sensory primordia at stage 13. (C, D) In dorsal views, *spg* is expressed in the brain and visceral mesoderm (arrowheads) at stage 13 (C) and brain and dorsal vessel at stage 16 (D, arrow). (E, F) At stage 16, expression is high in the ventral nerve cord in both lateral (E) and ventral (F) views. Arrow designates dorsal vessel expression (E). (G–I) Expression of Spg visualized by immunohistochemical staining. Spg is expressed is the ventral nerve cord in stage 13 (G) and stage 15 (H) embryos. Low expression is also detectable in the gut mesoderm (H). (I) A ventral view shows expression in the both the ventral nerve cord and peripheral neurons (arrows). (J-L″) Immunofluorescent confocal micrographs of Spg protein and neuronal markers. (J-J″) In stage 13 embryos, Spg expression overlaps with BP102 in both longitudinal and commissural axons. (K-K″) Spg is not expressed in repo (+) glial cells or ventral midline glial cells (L-L″). Anterior is left and dorsal is up in A, B, E, G, H. Scale bar = 50 µm.

Portions of the *spg* transcript were identified in a screen for neural precursor genes [52]. We confirmed this using *in situ* hybridization analysis that revealed *spg mRNA* is expressed strongly in the developing nervous system throughout embryonic development. *In situs* showed *spg mRNA* is detected in the nervous system primordia and sensory neurons in stage 11 and stage 13 embryos (Figure 2A, B). This strong expression persisted in the ventral nerve cord until the end of embryogenesis (Figure 2E, F). Staining in the visceral mesoderm in stage 13 embryos (Figure 2C, arrowheads) confirmed the identification of Spg from our muscle-specific MS analysis as the *mef2-GAL4* driver is expressed in both the visceral and somatic musculature. Similar to *mbc* [26], *spg mRNA* expression was also apparent in the dorsal vessel (Figure 2D, E, arrows). While *mbc* is also expressed abundantly in the developing somatic, or body wall musculature [26], *spg* expression is low or undetectable in this tissue (Figure 2C, solid lines). Thus, *spg* and *mbc* exhibit overlapping RNA expression patterns in the developing visceral musculature and dorsal vessel [26], while they are uniquely expressed in others. Mbc is strong in the somatic musculature, while Spg expression is predominant in the developing nervous system.

To confirm and extend our mRNA expression analysis, we examined the distribution of Spg protein using antisera generated against the C-terminal region of Spg. Consistent with *spg* mRNA expression, Spg protein was detected in the ventral nerve cord and visceral mesoderm (Figure 2G, H). A ventral view also revealed expression in the peripheral neurons (Figure 2I, arrows). In addition, Spg immunoreactivity was apparent in all longitudinal and commissural neurons (Figures 2J-J″). Spg was not detected in the general population of glial cells by co-staining with the glial cell marker Repo at stage 13 (Figure 2K-K″) or the midline glial cell marker Slit at stage 16 (Figure 2L-L″).

### Spg and ELMO are required for development of the central nervous system

All alleles of *spg* isolated in the laboratory of Christian Nüsslein-Volhard and analyzed by the Weischaus lab were homozygous viable and female sterile [48]. Although many of the original alleles were not available for these studies, a stop codon was identified by sequencing the *spg^242^* (previously called *spg^2^*) allele (W487*). Consistent with Postner, et al. [48], we found that eggs produced from *spg^242^* homozygous mothers with a mutant paternal allele of *spg* die early in embryonic development. To confirm that the lethality of *spg* is due to the *spg* locus, we were able to rescue this lethality by driving a *UAS-spg* cDNA with the early *nanos-GAL4* driver (n = 208). As maternal *spg* mutants die early and could not be examined for defects in later developmental processes, we examined embryos zygotically mutant for *spg^242^/spg^242^* for defects in nervous system development.

**Figure 3:**
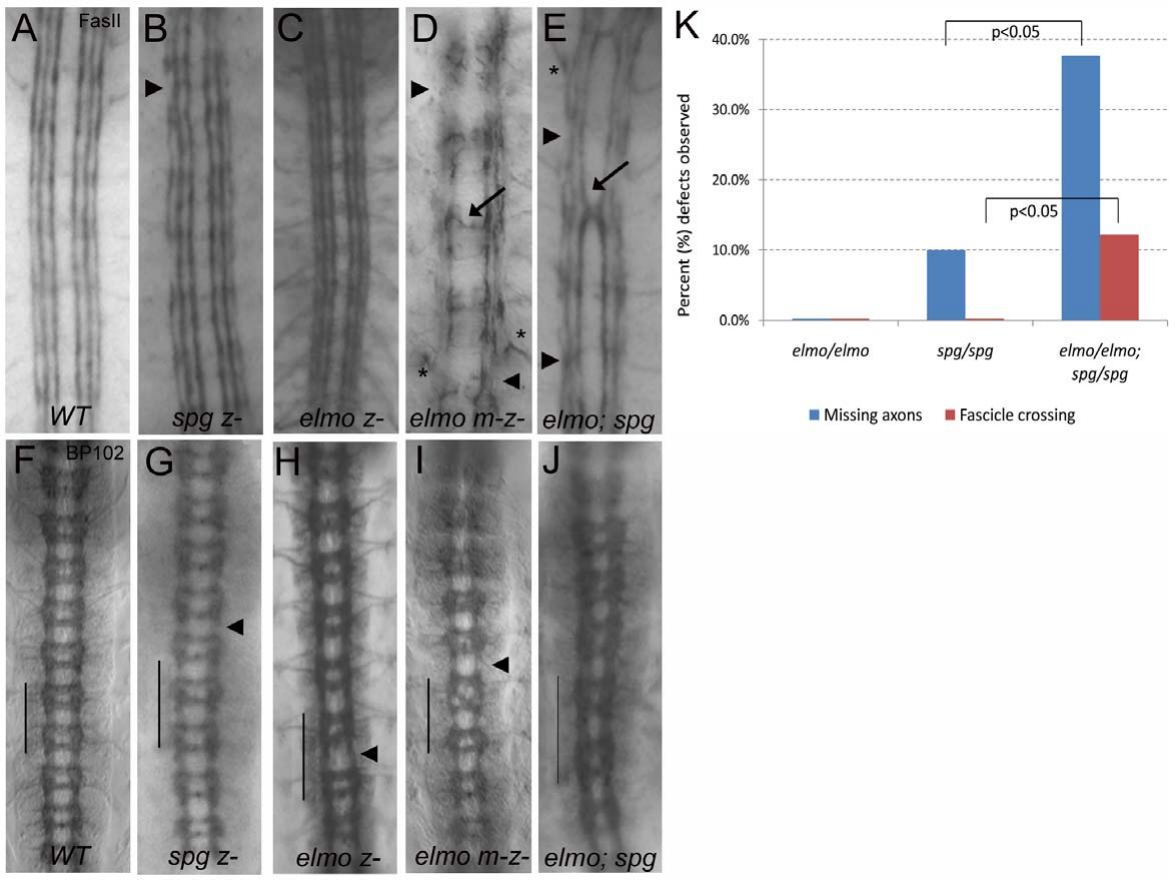
Embryos with loss of both zygotic *elmo* and *spg* exhibit abnormal axonal patterns. Late stage 16 or stage 17 embryos stained with anti-FasII to reveal subsets of longitudinal axons (A–E) and anti-BP102 to label all CNS axons (F–J). Anterior is up in all panels. (A, F) In WT embryos, FasII is expressed in 3 longitudinal bundles along each lateral side of the ventral nerve cord and BP102 labels both longitudinal and commissural axons on either side of the midline. (B, G) Removal of zygotic *spg* results in minor gaps in the outermost longitudinal fascicles (B, arrowhead) and a largely normal ladder-like pattern with occasional thinning of the longitudinal axons (G, arrowhead). (C, H) Embryos that lack zygotic *elmo* look similar to WT as visualized by anti-FasII (C) and reveal minor thinning of longitudinal axons with anti-BP102 (H, arrowhead). (D, I) Removal of maternal and zygotic *elmo* visualized by FasII (D) reveal discontinuous bundles of lateral axon tracts (arrowheads) and aberrant midline crossing of fascicles (arrow). Misrouted 1D4-positive axons are also seen outside the normal longitudinal pathways (asterisk). (I) Thinner longitudinal axons (arrowhead) and abnormal commissural patterns are present with BP102 in *elmo ^m-z-^* animals (I). (E) Analysis of embryos homozygous for both zygotic *elmo* and *spg* exhibit more severe axonal discontinuities and/or fusion to adjacent fascicles (arrowheads), in addition to inappropriate midline crossing (arrow). (J) These embryos also exhibit abnormal patterning of longitudinal and commissural axons (compare length of 2 consecutive segments denoted by line in J to F–I). (K) Graph depicting the percent of hemisegments that exhibit either gaps or missing axons and ectopic fascicle crossing in either *spg* or *elmo* mutants alone or *elmo*, *spg* double mutants. All embryos were stained with FasII for scoring (see table 1 for complete data set). Statistical significance was determined by student T-test.

For proper innervation of muscles in development, neurons send out actin-rich growth cones (outgrowth), bundle and unbundle when appropriate (fasciculation), and make decisions to cross the ventral nerve cord (axon guidance). For all experiments that include analysis of axon outgrowth and guidance, Fasciclin II (FasII) was utilized to label three tracts of longitudinal fascicles that run parallel to the nerve cord. A WT embryo labeled with FasII is shown in Figure 3A. Breaks in the longitudinal fascicles indicate axon stalling or outgrowth defects, while axons that cross the ventral midline are misguided. The global neuropile marker BP102 labels all longitudinal and commissural axons, resulting in a ladder-like appearance of the axonal projections (Figure 3F). Consistent with a maternal contribution of Spg mRNA and protein, embryos homozygous mutant for the *spg^242^* allele exhibited minor defects in the axonal patterns. Labeling with FasII revealed infrequent breaks in the outer longitudinal tract, while occasional thinning of these tracks were observed with BP102 (Figures 3B, G; Table 1). We could not address whether protein was reduced in *spg^242^* animals as the stop codon at AA487 truncates the protein before the region against which the Spg antibody was produced. Thus, we chose to analyze *spg^242^* over the deficiency line Df(3R)3450, which removes the *spg* locus [53]. In embryos of the genotype *spg^242^/Df(3R)3450*, we observed a similar percentage of gaps in the outer longtudinal fascicles to that of *spg^242^/ spg^242^* (Table 1). Furthermore, the frequency of outgrowth defects observed in *spg^805^/Df(3R)3450* and *spg^242^/ spg^805^* alleleic combinations were consistent (Table 1, Figure S1). To see if we could observe increased defects via neuronal-specific knockdown of Spg, we expressed *UAS-spg RNAi* using the pan-neuronal driver *C155-GAL4*. In addition to increased axon outgrowth defects (Table 1), we observed occasional bifurcated bundles, indicative of fasisculation or abnormal fusion defects (Figure S1E). The localization of *spg* expression in the developing nerve cord and Spg-ELMO complex based upon mass spectrometry results led us to examine the role of *elmo* genetically in development of the CNS. As predicted based upon the maternal contribution of ELMO mRNA and protein, embryos homozygous mutant for *elmo^19F3^*exhibited minor defects in axonal patterning. FasII labeling revealed a nearly wild-type pattern of all longitudinal fascicles, while occasional thinning of these tracks and increased length of adjacent segments were observed with BP102 (Figures 3C, H; Table 1). As described in Geisbrecht et al., this allele contains a stop codon at amino acid 393 and appears to be null as removal of both the maternal and zygotic contribution of *elmo* by germline clone analysis (GLC) resulted in early embryonic lethality [27]. Consistent with this, FasII staining in embryos homozygous for the deletion allele *elmo^ko^* [49] appeared normal (Table 2) and also resulted in early embryonic lethality when analyzed by GLC analysis. To reduce *elmo* function, yet allow animals to survive until the later stages of embryogensis when CNS development occurs, we used a hypomorphic *elmo* allele for GLC analysis [27]. In representative embryos maternally and zygotically mutant for *elmo^PB[c06760]^*, a dramatic increase in axonal patterning defects were observed. In addition to an increased number of outer fascicle gaps, we saw aberrant midline crossing of longitudinal axons, and misrouting of outer longitudinal axons (Figures 3D, I; Table 2). This suggests that *elmo* functions in CNS development in addition to its role in myoblast fusion and border cell migration [27], [49].

**Table 1:** *spg* alleles exhibit minor axonal outgrowth defects.

Genotype	OutgrowthDefects^a^	GuidanceDefects^b^	SegmentsScored (n)
*spg^242^/spg^242^*	10 (10.0%)	0 (0.0%)	100
*spg^242^/Df(3R)3450*	14 (14.8%)	2 (2.1%)	94
*spg^805^/Df(3R)3450*	21 (11.7%)	1 (0.0%)	179
*spg^242^/spg^805^*	28 (13.8%)	0 (0.0%)	202
*c155-GAL4/UAS-spgRNAi*	73 (28.0%)	0 (0.0%)	260

Stage 16–17 embryos stained with anti-FasII were scored.

a Scored as longitudinal axon tracts missing from either or both sides of nerve cord/segment.

b Normal fascicle(s) ectopically crossing the midline.

**Table 2:** Genetic interactions between *elmo*, *spg*, *mbc*, and *N-cad*.

Genotype	OutgrowthDefects^a^	GuidanceDefects^b^	% SegmentsAbnormal^c^	SegmentsScored (n)	% Embryos to severe to quantitate
*y, w*	0 (0.0%)	0 (0.0%)	0.0%	101	0.0% (n = 15)
*elmo^KO^/elmo^KO^*	1 (0.8%)	0 (0.0%)	0.8%	133	0.0% (n = 16)
*elmo^PB^m-z-*	35 (44.8%)	5 (6.4%)	72.0%	79	0.0% (n = 17)
*spg^242^/spg^242^*	10 (10.0%)	0 (0.0%)	10.0%	100	0.0% (n = 13)
*elmo^KO^/elmo^KO^*; *spg^242^/spg^242^*	40 (37.7%)**	13 (12.2%)**	50.0%	106	0.0% (n = 21)
*mbc^D11.2^/mbc^D11.2^*	23 (34.3%)	3 (4.4%)	38.8%	69	0.0% (n = 21)
*spg^242^, mbc^D11.2^/spg^242^, mbc^D11.2^*	97 (39.7%)	23 (9.4%)	49.1%	244	11.3% (n = 63)
*Ncad1^405^/Ncad1^405^*	24 (23.0%)	3 (2.8%)	25.9%	104	0.0% (n = 17)
*Ncad^Δ14^/Ncad^Δ14^*	81 (35.0%)	7 (3.0%)	38.0%	231	0.0% (n = 43)
*Ncad^Δ14^/+, spg^242^/spg^242^*	22 (40.0%)**	0 (0.0%)	40.0%	55	ND
*Ncad^Δ14^/Ncad^Δ14^*; *spg^242^/spg^242^*	97 (46.0%)**	48 (23.0%)**	69.7%	208	7.4% (n = 27)
*Ncad^Δ14^/Ncad^Δ14^*; *mbc^D11.2^/mbc^D11.2^*	115 (36.5%)	7 (2.2%)	38.7%	315	6.0% (n = 19)
*Ncad^Δ14^/Ncad^Δ14^*, *elmo^19F3^/elmo^19F3^*	133 (56.1%)**	10 (4.2%)	60.3%	237	4.7% (n = 63)

Stage 16–17 embryos stained with anti-FasII were scored.

a Longitudinal axon tracts missing from either or both sides of nerve cord/segment.

b Normal fascicle(s) ectopically crossing the midline.

c % segments abnormal includes all defects observed in a and b.

*m-z-* designates removal of maternal and zygotic contribution.

**indicates p<0.05 using student T-test compared to single mutants alone.

ND = not determined.

If two genes act in the same pathway, transheterozygosity for the two genes of interest may result in a phenotype stronger than the single mutants alone. This type of experiment is complicated in the case of *elmo* and *spg*, which are both contributed maternally. To examine if loss-of-function phenotypes could be exacerbated by removal of genes that function in the same pathway, zygotic embryos of the genotype *elmo^19F3^/elmo^19F3^*; *spg^242^/spg^242^* were analyzed. Compared to *elmo/elmo* (0.0%; n = 133) or *spg/spg* (10.0%; n = 100) single mutants, a consistent increase in longitudinal axon defects were observed in the double mutants (37.7%; n = 106; Table 2). In addition, we observed an increase in axons that inappropriately cross the midline (Table 2). A representative example is shown in Figure 3E and quantified in Figure 3K. By BP102 staining, abnormalities in the spacing between adjacent segments was also enhanced (Figure 3J). There are two possibilities to explain this result: (1) the double mutant is phenotypically stronger than either single mutant as the residual maternal products are compromised; or (2) the stronger phenotypes observed in the double mutant combination are a result of two pathways being affected. The two possibilities are not mutually exclusive. We favor the first hypothesis as we know Elmo-Spg are found in a complex based upon our MS and IP results. Furthermore, we do not observe genetic interactions with other candidates that may function with *elmo*.

### No muscle patterning defects are observed in mutants lacking Spg

**Figure 4:**
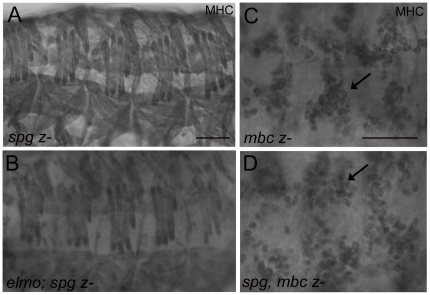
Loss of zygotic *spg* is not sufficient to reveal myoblast fusion defects. (A–D) Lateral views of stage 16 embryos stained with anti-MHC to visualize the final muscle pattern. (A, B) A wild-type muscle pattern is seen in mutants that lack zygotic *spg* (A) and both zygotic *elmo* and *spg* (B). (C, D) Myoblasts fail to fuse but cluster around founder cells (arrows) in *mbc* mutants (C) and *spg*, *mbc* double mutants (D). Scale bar = 10 µm.

Based upon the complementary expression patterns for *mbc* and *spg* in the somatic musculature and developing CNS, respectively, an attractive notion would be that ELMO binds to and functions with Mbc and Spg in a tissue-specific manner. To explore this, we examined phenotypes of single and/or double mutants in both muscle and nervous system development. Consistent with our above results that removal of zygotic *spg* exhibited almost wild-type axonal patterning, no myoblast fusion defects were observed in zygotic *spg^242^/spg^242^* mutant embryos (Figure 4A). In addition, we did not observe unfused myoblasts just under the somatic muscle layer (data not shown). In contrast to defects observed in the CNS in *elmo*; *spg* double mutants, analysis of the final muscle pattern in these embryos appeared wild-type (Figure 4B). As previously reported, loss-of-function mutations in *mbc* resulted in strong myoblast fusion defects in the developing embryo [11], [26]. In homozygous embryos mutant for *mbc^D11.2^*, the myoblasts were competent to migrate to the founder cells where fusion normally takes place, while fusion did not occur (Figure 4C). To examine if *spg* may be functioning redundantly with *mbc* in myoblast migration, the distribution of myoblasts was examined in *mbc^D11.2^*, *spg^242^/mbc^D11.2^*, *spg^242^* double mutants. While the myoblasts fail to fuse as in *mbc* mutants, they were still capable of clustering around the founder cells, suggesting that myoblast migration was not affected (Figure 4D).

### Both Spg and Mbc are required for axonal patterning

**Figure 5:**
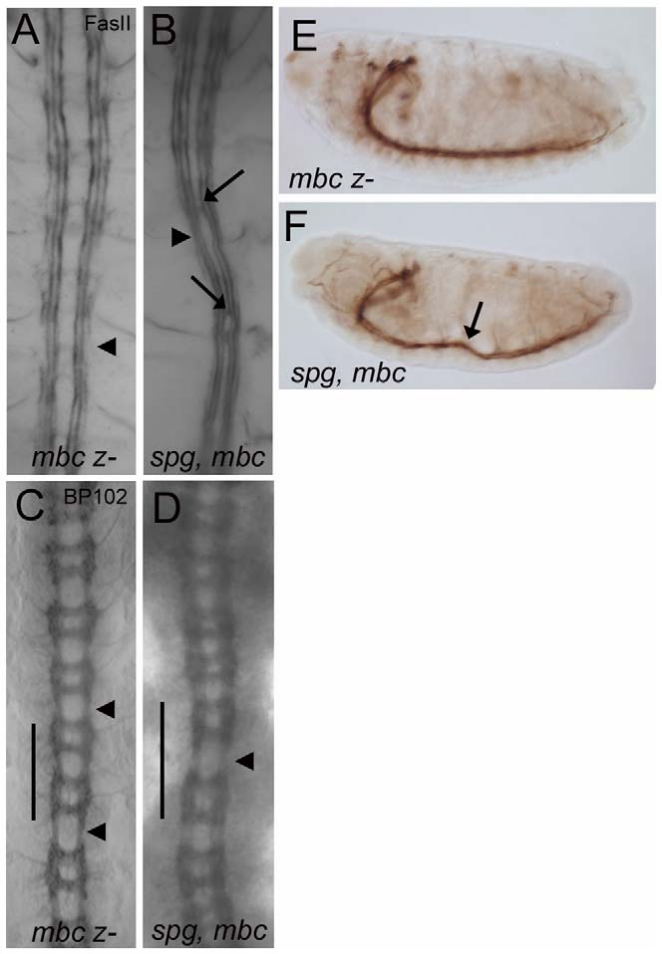
CNS defects are enhanced in embryos missing both *spg* and *mbc*. Late stage 16 or stage 17 embryos stained with anti-FasII (A, B, E, F) and anti-BP102 to label all CNS axons (C, D). (A, C) *mbc* mutants have more discontinuities in the outermost fascicles (A, arrowhead) and thinner longitudinal axons (C, arrowheads). (B, D) Mutants missing both *spg* and *mbc* have an increase in (B) missing and collapsed longitudinal fascicles (arrowhead) and abnormal crossovers (arrows). BP102 staining (D) shows a severe thinning of axons (arrowhead) and abnormal spacing between segments (compare length of 2 consecutive segments denoted by line in panels C and D). (E, F) Lateral views of stage 16 embryos stained with anti-FasII show abnormal positioning of the ventral nerve cord in *spg*, *mbc* mutants (F, arrow) compared to *mbc* mutants alone (E). Anterior is up in panels A–D. Anterior is left and dorsal is up in panels E, F.

The experiments above indicate Spg is not required in embryonic muscle development. To further examine if Spg is the only DOCK family member required for axonal patterning, we examined the potential contribution of Mbc in the developing nervous system. Similar to defects already observed in *spg* mutants, embryos homozygous mutant for *mbc^D11.2^* exhibited breaks in the outer longitudinal fascicles (Figure 5A; Table 2). In addition, we observed collapse of axons onto the MP1 fascicle tracts (data not shown). This extends and supports observations by Nolan, et al., where it was determined that embryos transheterozygous for *mbc^1.63^*/*mbc^4.25^* exhibited ventral nerve cord defects upon examination with BP102 [32]. Our analysis using BP102 phenocopies their results, where we observed thinning of the longitudinal axon tracts and abnormal spacing between segments (Figure 5C). This suggests that low expression of *mbc*, possibly undetected in the CNS due to high expression in the muscle, contributes to nervous system formation.

As Spg and Mbc are the two DOCK family members predicted to be specific for Rac and mutations in either one exhibit defects in the nervous system, we sought to examine if embryos mutant for both *mbc* and *spg* resulted in enhanced nervous system defects. We did not observe a significant increase in broken fascicles or the collapse of the outer longitudinal tracts in *mbc*, *spg* double mutants over *mbc* mutants alone (Figure 5B, Table 2). However, we did observe an increase in midline fascicle crossing in these double mutants (Figure 5B, arrows, Table 1). There was also an increase in abnormal positioning of the ventral nerve cord in *mbc*, *spg* double mutants, where 48.2% of mutant embryos (n = 56) exhibited abnormal swerving of the nerve cord seen on the ventral side (Figure 5B, 5D) or abnormal bends in lateral views (Figure 5F compared to Figure 5E), which was rare in single mutants of *spg* (0.0%; n = 23) or *mbc* mutants (0.8%; n = 22). The above data suggests Mbc may be the primary DOCK family member in tissues like the muscle, while both Spg and Mbc may function in other tissues, such as CNS development and border cell migration.

### Expression of N-cadherin is sufficient to recruit Spg to the membrane in S2 cells

**Figure 6:**
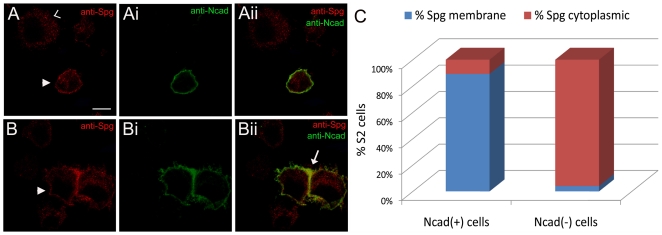
Expression of N-cadherin is sufficient to recruit Spg to the membrane. (A-Bii) Confocal micrographs of S2 cells transfected with Ncad and stained for Ncad (green) to detect transfected cells and endogenous Spg (red). (A-Aii) In a singly transfected cell, Spg is recruited to the membrane (closed arrowhead) compared to untransfected cells where Spg is cytoplasmic (open arrowhead). (B-Bii) Homotypic cell adhesion between two Ncad-expressing S2 cells also results in apparent membrane Spg staining (closed arrowhead), most notably at sites of cell-cell contact between adjacent cells (arrow). (C) Quantification of Spg subcellular localization in cells either transfected with or without Ncad. The percentage of S2 cells were scored for either membrane or cytoplasmic Spg localization. Scale bar = 5 µm.

Scanning through our list of potential MS candidates, N-cadherin (Ncad) emerged as a possible upstream receptor to mediate signaling via DOCK-ELMO complexes, albeit at low levels. Furthermore, Ncad is expressed in the embryonic fly nervous system and vertebrate MOCA/DOCK3 colocalizes with Ncad in regions of cell-cell contact in the nerve cell line PC12 [40], [54]. Thus, Ncad seemed a reasonable candidate to examine it's involvement with DOCK-ELMO complexes in CNS development. To gain insight into a potential Ncad-Spg interaction, we examined the subcellular distribution of Spg and Ncad protein in *Drosophila* S2 cells. RT-PCR results show that *spg* is endogenously expressed in S2 cells (data not shown). Furthermore, staining with anti-Spg antibody reveals a cytoplasmic localization of the protein (Figure 6A). As S2 cells do not endogenously express *Ncad*, cells transfected with full-length Ncad were stained for Ncad and Spg protein. In transfected cells, Ncad was detected at the membrane and was capable of aggregating with other Ncad(+) cells (Figure 6B′), a hallmark of the homotypic cell adhesion properties of the Cadherin family of proteins [54]. The subcellular distribution of Spg was cytoplasmic in Ncad(−) cells (Figure 6A, 6A″, 6B, 6B″), but became membrane localized upon expression of Ncad (Figure 6A′, 6A″). In Ncad(+) cells that formed clusters, Spg localization was enriched at the membrane between adjacent cells (Figure 6B′, 6B″). To quantify these observations, we acquired confocal images of S2 cells both with and without Ncad expression. As shown in Fig. 6, we observed membrane-enriched Spg in 89.2% of cells (n = 102) of Ncad (+) cells compared to 0.04% of S2 cells that do not express Ncad (n = 210).

### Genetic analysis of Ncad-Spg mutants

**Figure 7:**
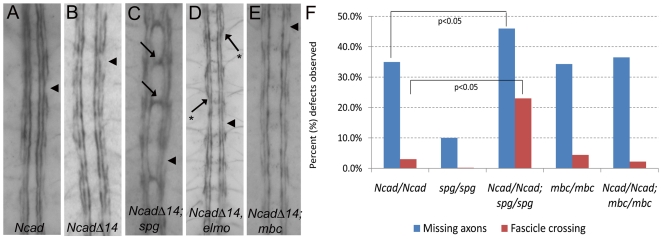
Genetic interactions between *Ncadherin*, *elmo*, *spg*, and *mbc*. (A–E) Anti-FasII staining to visualize longitudinal axons. (A, B) Removal of zygotic *Ncad* (A) or both N-cadherin genes (*Ncad^Δ14^*) (B) exhibit mild axonal break defects (arrowheads). (C) A significant increase in both fascicle axonal breaks (arrowhead) and ectopic midline crossing (arrows) are observed in *Ncad^Δ14^*; *spg* double mutants. (D) Removal of both *Ncad^Δ14^* and *elmo* function results in an increase in axonal patterning defects, including a collapse of the outer fascicle tract onto the MP1 fascicle (asterisk and arrow) and an increase in axonal gaps (arrowhead). (E) *Ncad^Δ14^*; *mbc* double mutants exhibit many breaks in the outer longitudinal fascicles (arrowhead), similar to that of *Ncad^Δ14 ^*or *mbc* alone. (F) Graph showing the percent of hemisegments that exhibit missing axons or ectopic fascicle crossing in *Ncad^Δ14^*, *spg*, or *mbc* single and double mutants. A statistically significant difference (using student t-test) is observed in *Ncad^Δ14^*; *spg* double mutants versus the *Ncad^Δ14 ^*or *spg* single mutants alone. However, analysis of double mutants of *Ncad^Δ14^*; *mbc* do not show a significant increase in axonal defects over the single mutants alone.

Based upon the results that Spg is enriched at the membrane upon expression of Ncad in S2 cells, we wondered if removal of Ncad could increase the severity of *spg^242^/spg^242^* axonal phenotypes. As previously reported for other *Ncad* alleles, mutants for *Ncad^405^/Ncad^405^(Ncad)* alone show mild CNS defects (Figure 7A; Table 2) [54]. The Clandinin lab created mutants that remove both *Ncad* and the recently characterized *N-cadherin2 (Ncad*, *Ncad2* double mutant, hereafter called *Ncad^Δ14^*) [55]. Thus, we examined *Ncad^Δ14^*mutants to determine if these proteins may function redundantly in CNS development. It appears the contribution of *Ncad2* is minor or negligible as our results do not show quantifiable differences between *Ncad* mutants alone or *Ncad^Δ14^*/*Ncad^Δ14^* double mutants (Table 2). Removal of one copy of *Ncad^Δ14^* in a *spg^242^/spg^242^* homozygous mutant background increased the occurance of axon outgrowth defects over *spg^242^* mutants alone (Table 2). To examine this further, we also quantitated embryos double mutant for both *Ncad^Δ14^* and *spg^242^*. We observed a modest, although significant increase in axon outgrowth phenotypes over *Ncad^Δ14^* mutants alone (Figure 7C, Table 2). Consistent with this, *Ncad^Δ14^*, *elmo^19F3^* double mutants exhibited a consistent enhancement of axonal breaks (Figure 7D, Table 2), although no increase in midline guidance errors. However, in both double mutant combinations, we also observed qualitatively different and/or stronger phenotypes than that observed in the single mutants alone. For example, we also observed a greater than additive increase in ectopic midline crossing in *Ncad^Δ14^*; *spg^242^* double mutants (23.0%) over *Ncad^Δ14^* (3.0%) or *spg^242^* (0.0%) mutants alone. In *Ncad^Δ14^*, *elmo^19F3^* double mutants, the embryos showed an increase in collapsed outer longitudinal axon tracts onto the MP1 fascicle (Figure 7D, asterisks), a phenotype not observed in *Ncad^Δ14^* or *elmo^19F3^* mutants alone. These data taken together suggest that the maternal load of *spg* or *elmo* may be masking phenotypes until the levels of an upstream component is compromised. An alternative explanation is that Ncad, Spg or Elmo may also have functions independent of one another in CNS development. Although *mbc* is required for axon outgrowth (Figure 5A), we did not observe an increase in axonal outgrowth or guidance defects upon removal of Ncad (Figure 7E), suggesting that Mbc may function independently.

## Discussion

Recent investigations of vertebrate DOCK family proteins demonstrate that DOCK-ELMO complexes function together to regulate downstream GTPases, namely Rac. In this study, we uncover the *Drosophila* DOCK family member Spg, and find that mutations in *elmo*, *spg*, or *mbc* exhibit abnormal axonal patterning in the embryonic CNS. Ncad is capable of relocating cytosolic Spg to the membrane in S2 cells. Furthermore, we found that mutations in *Ncad* dominantly enhance the axonal outgrowth phenotypes of *spg* mutants and that *Ncad*; *spg* and *Ncad*, *elmo* double mutants have more severe CNS phenotypes. Taken together, these data indicate (1) that Ncad, Spg, and Elmo may function together during axonal outgrowth, (2) that the severe double mutant phenotypes reflect a decrease in the function of maternally loaded components that were masked in single mutants, and/or (3) the double mutant defects represent a disruption of multiple signaling pathways.

### Identification and characterization of Spg, a DOCK family member

We identified peptides corresponding to the uncharacterized protein CG31048 in an *in vivo* mass spectrometry approach to identify ELMO-binding partners. The *CG31048* locus, which encodes for Sponge, is a member of the growing family of *Drosophila* DOCK family proteins. This report is the second identification of a DOCK family member in flies since the role of Mbc was uncovered in 1997 [26]. The 11 vertebrate DOCK proteins identified thus far can be divided into subgroups based upon primary sequence analysis and GTPase target specificity for either Rac or Cdc42 [5], [10], [50]. In the first group, the DOCK-A family consists of DOCK180, DOCK2, and DOCK 5, while the DOCK-B subfamily is comprised of DOCK3 and DOCK4. In flies, this redundancy is simplified with the 2 DOCK family members, Mbc and Spg, whom are members of the DOCK-A and DOCK-B groups, respectively. All of the above family members contain an N-terminal SH3 domain, 2 internal DHR (CZH) domains and a variable C-terminal proline-rich region. Furthermore, they function as unconventional guanine nucleotide exchange factors (GEFs) for the GTPase Rac. Members of the DOCK-C (DOCK 6, DOCK7, DOCK8) subfamily and DOCK-D (DOCK9, DOCK10, DOCK11) subfamily bind to the GTPase Cdc42. The 2 orthologous Drosophila proteins, CG42533/Dm ziz (DOCK-C) and CG11376/Dm zir (DOCK-D) have not yet been characterized in flies.

Alleles of *spg* were originally identified in a maternal effect screen and later characterized for their role in actin-dependent events in early *Drosophila* embryogenesis [47], [48]. Our mRNA and protein expression analysis suggested Spg may be required after cellularization due to strong expression in the visceral mesoderm, dorsal vessel, and developing ventral nerve cord. As removal of the maternal contribution of *spg* null alleles results in early embryonic lethality, the role for *spg* in later developmental processes had not been examined. However, the identification of Spg as an ELMO-interacting protein gave us insight into how to examine the role of Spg in late embryogenesis using double mutant analysis. While zygotic single mutants of *spg* and *elmo* appeared essentially wild-type, removal of both the zygotic contribution of both *spg* and *elmo* resulted in axonal patterning defects. We favor the hypothesis that the maternal contribution of both Elmo and Spg mask any embryonic phenotypes until the levels of both proteins are compromised. Alternatively, though not mutually exclusive, is the possibility that Elmo and Spg function in parallel pathways and our observed phenotypes are a result of these additive effects. As mentioned above, removal of either *spg* or *elmo* maternal contribution results in early embryonic lethality [27]. As *spg* has shown to be required for early actin cap and metaphase furrow formation, it is fair to hypothesize that that these two genes may function in concert in early embryo development, where Mbc is not required.

### Downstream GTPase of the DOCK-ELMO complexes

**Figure 8:**
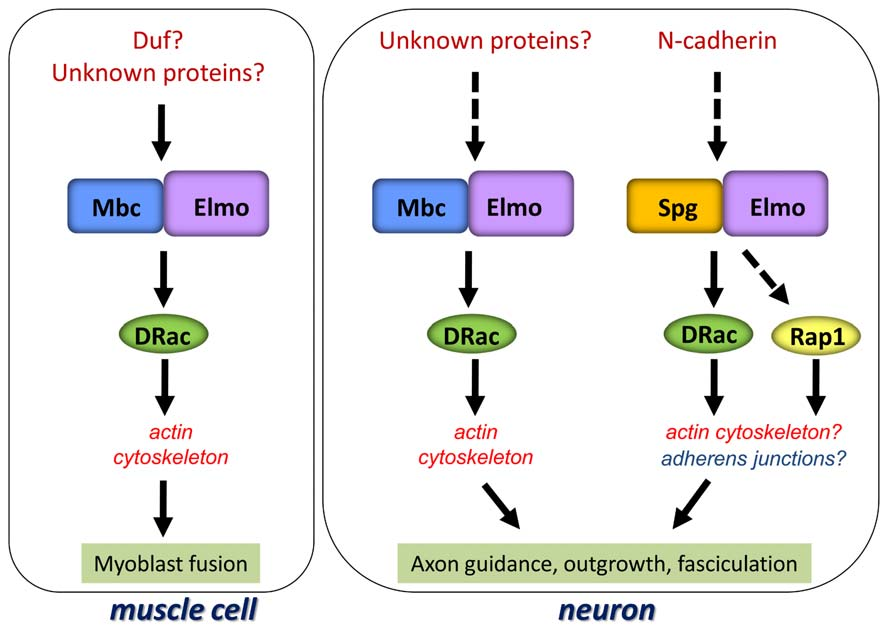
Model of CDM-Elmo pathway. In the muscle, Mbc is the sole CDM family member that functions with Elmo to mediate cytoskeletal modifications through the GTPase Rac (left panel). In a neuronal cell (right panel), both Mbc and Spg contribute to nervous system formation. In this model, the Mbc-Elmo complex is downstream of yet unidentified proteins and presumably signals through Rac. In contrast, our data suggests Spg-Elmo may function downstream of Ncad. The target of the Spg-Elmo complex, whether it be Rac and/or Rap is unclear.

Vertebrate DOCK 4 was originally identified as a CDM family member capable of activating the small GTPase Rap1 in GTPase pull-down assays [56]. Functionally, a deletion of endogenous DOCK4 in osteosarcoma cells was shown to rescue the formation of adherens junctions and could be suppressed by co-expression of dominant-negative Rap1 [56]. Recent studies have demonstrated that DOCK 4 is also capable of activating the GTPase Rac1 [45], [51], [57]. This data suggests that GTPase activation of either Rac and/or Rap1 by the Spg-ELMO complex is context and/or tissue-dependent. Our current model for DOCK-ELMO function in embryogenesis is shown in Figure 8. Only the Mbc-ELMO complex functions in the developing musculature to activate the GTPase Rac. While it is clear that regulation of the actin cytoskeleton is downstream of the Mbc-ELMO→Rac signaling pathway, the upstream receptors that mediate this signaling are unknown. Our data suggests that both Mbc and Spg function in the *Drosophila* developing nervous system. All literature thus far supports a model whereby the Mbc-ELMO complex activates Rac. Alternatively, the Spg-ELMO complex may regulate Rac and/or Rap1 activity. If both the Mbc-ELMO and Spg-ELMO protein complexes function upstream of Rac, they may be acting redundantly to regulate Rac-dependent actin cytoskeletal changes. Alternatively, the downstream effector functions of Rac activity may lead to changes in cell-cell adhesion or may be mediated through the GTPase Rap1. We hypothesize that differences in the C-terminal proline-rich regions of Mbc and Spg may be responsible for their differential activities. In myoblast fusion, the proline-rich region of Mbc is not required [11]. However, Spg and vertebrate DOCK3/4 contain additional proline-rich sites not present in Mbc/DOCK180. Further experiments will be necessary to define the cellular and molecular mechanisms necessary to carry out DOCK-ELMO functions in the developing CNS.

### Regulation of GEF activity

ELMO expression is ubiquitous throughout fly development, while Mbc and Spg expression is predominant in the muscle and nervous system, respectively. Based upon the tissue-specific expression patterns of Mbc and Spg, we originally hypothesized that complementary expression patterns may be one mechanism for the tissue-specific regulation of Rac activation through the DOCK-ELMO complexes. However, our results indicate that the role of Mbc-ELMO and Spg-ELMO is more complicated. While the Mbc-ELMO complex seems to be the primary GEF complex for Rac activation in the musculature, both the Mbc-ELMO and Spg-ELMO complexes may both be necessary to correctly pattern axons in the developing central nervous system. In support of the idea that both complexes are required in certain developmental situations, the Rorth lab found that both Spg and Mbc are required in border cell migration [49]. Removal of both Spg and Mbc function in the border cells phenocopies loss of ELMO, suggesting that these 2 genes function in concert with ELMO to guide migration. Further experiments are required to determine if the observed CNS defects in *spg* and *mbc* mutants are autonomous in the nervous system. Alternatively, axonal patterning defects observed in *mbc* mutants may be a secondary consequence due to a requirement for Mbc in the musculature.

In the musculature, the only known GEF shown to be required for Rac activation is the Mbc-ELMO complex. However, in the developing nervous system, in addition to the unconventional DOCK-ELMO complexes, the conventional GEFs Trio and Sos are required [9], [58], [59], [60], [61]. It is not clear how these multiple GEFs are regulated throughout CNS development. Possible mechanisms include the: (1) regulation of GEF expression either in subsets of specific neurons or precise subcellular localization within the same neuron; (2) unique physical associations between GEFs and receptors specific for distinct steps in axonal patterning; and (3) regulation of GEF activity via post-translational modifications including phosphorylation or ubiquitination. While these ideas have not been examined in detail for all known GEFs, what is known is discussed below.

First, it is possible mechanisms exist within the cell or tissue to compartmentalize GEF function as the spatial expression patterns of all GEFs in the developing ventral nerve cord seems to be fairly broad. Mbc is expressed at low or undetectable levels with reagents currently available, while Spg is expressed in all commissural and longitudinal axons, but not glial cells. Likewise, Sos protein is broadly expressed in many cell types around stage 12 and becomes enriched in CNS axons [9]. While Trio is expressed in axons that run on longitudinal tracts and those that cross the midline, enrichment of this protein is evident in the longitudinal fascicles [58]. Trio is largely localized near the membrane [62], while cytoplasmic Spg and Sos can be recruited to the membrane by their association with N-cadherin and Robo, respectively [9]. It is not yet clear if membrane recruitment is sufficient to promote Rac activation, or if conserved mechanisms exist to activate GEFs where their activity may be needed. For example, by binding to RhoG, ELMO can target DOCK180 to the membrane [17]. In addition, ELMO binding to DOCK180 relieves a steric inhibition by exposing the DHR-2 domain of DOCK180 that binds Rac [16]. This remains to be shown for other DOCK family members.

Next, it is possible that each distinct step of neuronal pathfinding requires a unique set of proteins that allow upstream receptors to signal to downstream proteins for a specific biological output. For example, Trio cooperates with the Abelson tyrosine kinase (Abl) to promote Rac-dependent actin cytoskeletal dynamics in Frazzled-mediated commissure formation [8]. In the separate process of longitudinal fascicle formation, a trimeric complex of Robo-DOCK-Sos activates Rac to promote axon repulsion [9]. Separately, N-cadherin is suggested to be required for fasciculation and directional growth cone migration [54]. Thus, the Ncad-DOCK-ELMO complex may be responsible for this latter aspect of axonal pathfinding, while other steps may be mediated by individual receptor-GEF complexes. However, additional evidence suggests this regulation may be more complex. Preliminary data from our laboratory demonstrates that Ncad may genetically interact with other Rac GEFs to affect earlier CNS development *Ncad* mutants cannot be rescued by expression of RacWT alone in the CNS (Biersmith, B. and Geisbrecht, E.; unpublished data). DOCK180 binds the vertebrate receptor Deleted in Colorectal Cancer (DCC) (similar to the Netrin receptor Fra in flies) [63]. In addition, inhibition of DOCK180 activity decreased the activation of Rac1 by Netrin [37]. Another study suggests that Robo is required for multiple, parallel pathways in axon guidance and activated Robo function inactivates N-cadherin-mediated adhesion [63]. Current models suggest activated Robo binds to Abl and N-cadherin, thus providing a mechanism to weaken adhesive interactions during fasciculation to allow for mediolateral positioning of axons along the ventral nerve cord. The association of either Mbc or Spg proteins in the Netrin signaling pathway has not been examined. So far, we have not observed significant differences in genetic combinations that remove either *robo* or *slit* in *elmo* mutants (Lui, Z. and Geisbrecht, E.; unpublished data). Furthermore, no significant increases in midline guidance errors were observed in *Ncad*, *elmo* mutants, suggesting that Ncad and Spg may function in this process independent of ELMO function. It is clear that additional analysis of Robo and N-Cadherin dynamics are needed in the well-established CNS fly model to determine their *in vivo* relevance.

Finally, the physical interactions of GEF proteins with specific membrane receptors may allow the GEFs to be in a unique subcellular localization for post-translational modifications that regulate activity. As mentioned above, DOCK180 is capable of binding and activating Rac when sterically relieved upon ELMO binding [16]. In addition, the presence of ELMO1 inhibits the ubiquitination of DOCK180, thus stabilizing the amount of GEF available to activate Rac [64]. Finally, although the significance is unclear, DOCK180 is phosphorylated upon Integrin binding to the extracellular matrix [65]. Trio has also been shown to be tyrosine phosphorylated upon co-expression with Abl [8], suggesting this may be a common mechanism for GEF regulation. ELMO is also phosphorylated on tyrosine residues [66], providing another level of GEF regulation. Further experimentation must be done to determine whether these modifications of GEFs also lead to regulation of Rac activity.

## Materials and Methods

### Genetics

Fly stocks were raised on standard cornmeal medium at 25°C unless otherwise indicated. Oregon R was used as the wild-type strain. The following alleles/fly stocks were used: *elmo^19F3^, P{ry[+7.2] = neoFRT}40A* (Geisbrecht, et al, 2008); *elmo^PB[c06760]^*, *P{ry[+7.2] = neoFRT}40A* (Geisbrecht, et al, 2008); *elmo^KO^* (Bianco, et al, 2007); *spg^242^* and *spg^805^* (kindly provided by Eyal Schejter); *mbc^D11.2^* (Erickson, et al, 1997); *Ncad1^omb405^* (Yonekura, et al, 2007); *Ncad^Δ14^* (Prakash, et al., 2005). *elmo^PB.mat^* mutants were created as previously described (Geisbrecht, et al, 2008). The following stocks were generated by standard meiotic recombination and isolated on the basis of their failure to complement other alleles and/or sequencing to verify the molecular lesion: *Ncad^Δ14^*; *elmo^19F3^*; *spg^242^*, *mbc^D11.2^*. Additional stocks were generated by standard fly crosses: *elmo^KO^*; *spg^242^* and *Ncad^Δ14^*; *mbc^D11.2^. C155-GAL4* and *nanos-GAL4* were obtained from the Bloomington Stock Center and *UAS-spgRNAi* flies were obtained from the Vienna *Drosophila* RNAi Center (VDRC).

### *In situ* hybridization and immunostaining

Embryos were collected on agar-apple juice plates and aged at 25°C. For *in situ* analysis, multiple internal sequences encoding *spg* were transcribed with Sp6 using the DIG mRNA labeling kit (Roche) and hybridized as described [27]. For immunohistochemistry, embryos were fixed and stained as described [27]. The musculature was visualized using anti-MHC (1∶500). The CNS was labeled using mAb 1D4 (1∶100, Developmental Studies Hybridoma Bank, University of Iowa) and mAb BP102 (1∶20, Developmental Studies Hybridoma Bank, University of Iowa). Secondary antibody was goat anti-mouse-HRP (1∶200, Jackson). Fluorescent immunostaining was performed as previously described in Geisbrecht, et al [27]. Primary antibodies used were anti-Repo (1∶50, Developmental Studies Hybridoma Bank, University of Iowa) and anti-Slit (1∶50, Developmental Studies Hybridoma Bank, University of Iowa) and detected fluorescently using Alexa Fluor 488 goat anti-mouse IgG at 1∶400 (Molecular Probes, Carlsbad, CA). Tyramide staining was used to enhance Spg signal for immunofluorescent stainings (Vector Labs, Burlingame, CA).

### Mass spectrometry identification and immunoprecipiations

Mass spectrometry experiments were described previously [27]. For immunoprecipitations, ELMO-HA-tagged and untagged transgenic flies were crossed to *mef2-GAL4* females and 6–18h embryos were collected on agar-apple juice plates at 25°C. Embryos were dechorionated and homogenized in lysis buffer [60mM Tris (pH 7.5), 80mM NaCl, 6mM EDTA (pH 8.0), 2% Triton X-100, 1mM Na_3_VO_4_, 5mM 1-Naphthyl phosphate potassium salt, 2mM PMSF, 2 ug/ml Leupeptin, 2 ug/ml Pepstatin]. The NaCl concentration was increased to 300mM and resulting lysate mixed with anti-HA resin overnight at 4°C. The resin was washed 3 times with wash buffer plus protease inhibitors, boiled in 6× sample buffer and submitted to SDS-PAGE and subsequent Western blotting. The following primary antibodies were used for immunoblotting: anti-Spg (1∶1000, this paper), anti-ELMO (1∶1000) and anti-HA-HRP (1∶2000, Roche). After incubation with goat anti-guinea pig-HRP (Jackson), proteins were visualized with ECL Plus (Amersham).

### Constructs and Spg antibody production

A full length *spg* cDNA sequence was generated by analyzing multiple, overlapping fragments generated by RT-PCR using S2 cells and 0–6 h embryos as a reference source. A full length cDNA was generated by Epoch Biolabs and cloned into pUAST. Transgenic flies were produced by Genetic Services, Inc. using standard techniques. By standard RT-PCR techniques, gene-specific primers were used to amplify the region of *spg* corresponding to AA 1669–2023. The forward and reverse primers were engineered to contain SalI and NotI restriction sites, respectively. This cDNA fragment was cloned into the pT7MHT expression vector and soluble protein was purified as described [67]. This soluble protein was sent to Pocono Rabbit Farm and Laboratory Inc. for injection into guinea pigs. The resulting antisera was used at 1∶500.

### S2 cell transfections

Transient calcium phosphate transfections of pRmHA3_N-cadherin were carried out with 1.2×10^6^ cells/ml and 7–15 ug DNA as needed. Cells were induced 24 hours after transfection with 0.7 µM CuSO_4_. After 48 hrs, cells were resuspended at a concentration of 1.2×10^6^ cells/ml in 2 mls of BBS buffer (10mM HEPES, 55 mM NaCl, 40mM KCl, 15 mM MgSO_4_, 20 mM glucose, 50 mM sucrose, and 10 mM CaCl_2_). The cells were agitated in a 35 mm dish at 100 rpm for 1 hr. The cells were plated on poly-L-lysine coated coverslips and fixed for 10 minutes in 4% PFA in Ca^2+^ and Mg^2+^-free (CMF) C & GBS (55 mM NaCl, 40 mM KCl, 10 mM Tricine (pH = 6.9), 20 mM glucose, 50 mM sucrose)+1 mM CaCl_2_. Standard immunofluorescent protocols were followed using rat anti-Ncad (1∶20, Developmental Studies Hybridoma Bank, University of Iowa) and gp anti-Spg (1∶500). Secondary antibodies used were Fluor 488 goat anti-rat IgG and Fluor 546 goat anti-guinea pig at 1∶400 (Molecular Probes, Carlsbad, CA).

## Supporting Information

Figure S1 **Loss of Spg results in mild CNS defects.** (A–E) Stage 16 embryos stained with FasII. (A, B) Both *spg^242^* (A) and *spg^805^* (B) over a deficiency that removes the *spg* locus result in mild gaps in the outer longitudinal fascicles (arrowheads). (C) The same phenotype are observed in animals trans-heterozygous for *spg^242^* and *spg^805^*. (D, E) Knockdown of Spg by RNAi resulted in similar axonal outgrowth phenotypes (D) and bifurcated axons (asterisk in E).
(EPS)
